# Assessment of IL-6, IL-8 and TNF-α levels in the gingival tissue of patients with periodontitis

**DOI:** 10.3892/etm.2013.1222

**Published:** 2013-07-15

**Authors:** MIN KI NOH, MIN JUNG, SOO HWAN KIM, SEO RA LEE, KI HO PARK, DONG HWAN KIM, HANNA HYUN KIM, YOUNG GUK PARK

**Affiliations:** 1 Department of Orthodontics, School of Dentistry, Kyung Hee University, Seoul 130-701, Republic of Korea; 2 College of Arts and Science, New York University, New York, NY 10012, USA

**Keywords:** interleukin-6, interleukin-8, tumor necrosis factor-α, periodontitis

## Abstract

In periodontal diseases, inflammatory mediators, including interleukin (IL)-6, IL-8 and tumor necrosis factor-α (TNF-α), may promote the degeneration of inflamed periodontal tissues. In previous studies, levels of these three cytokines were demonstrated to be elevated in inflammatory gingival tissues and gingival crevicular fluid. The aim of the present study was to quantify IL-6, IL-8 and TNF-α levels in the human gingival tissues of patients with periodontitis and to assess the correlation of these three cytokines with each other. In this study, human gingival tissues from 19 patients with periodontitis (male, n=14; female, n=5) were collected. The tissues were homogenized, centrifuged and the protein in the supernatant was quantified. Enzyme-linked immunosorbent assay (ELISA) was used in the measurement of the IL-6, IL-8 and TNF-α levels, and the mean levels were observed to be 8.41±0.25, 34.01±1.09 and 20.70±0.31 pg/ml, respectively. The mean levels of IL-8 were higher than those of the other two cytokines. In each sample, the level of TNF-α expression was consistently high, with little difference between the results, which contrasted with the fluctuations in IL-6 and IL-8 levels. The expression of the two ILs (IL-6 and IL-8) showed a positive correlation (r=0.932, P=0.01), whereas TNF-α levels were not correlated with IL-6 or IL-8 levels. These results suggest that IL-6, IL-8 and TNF-α may be relevant in the pathophysiology of periodontitis, and the measurement of these cytokines may be beneficial in the identification of patients with periodontitis.

## Introduction

Human periodontal diseases are predominantly caused by infections with gram-negative bacteria, such as *Porphyromonas gingivalis* and *Bacteroides forsythus*(1). A complex interaction between these bacteria and the host immune system may induce inflammatory conditions that result in the loss of the collagenous structures that support the teeth (2,3). A number of inflammatory mediators, such as interleukin (IL)-1, IL-6, IL-8, tumor necrosis factor-α (TNF-α), prostaglandins and matrix metalloproteinases (MMPs) are involved in periodontal diseases (4,5). These mediators may affect the activities of leukocytes, osteoblasts and osteoclasts and promote the tissue remodeling process systemically and locally (6–8). The collagenolytic enzymes, including MMPs, are mediated by a variety of inflammatory cytokines, such as IL-1, IL-6, IL-8 and TNF-α (9,10).

IL-6, a multifunctional cytokine, has a number of biological activities, including B-lymphocyte differentiation, T-lymphocyte proliferation and the stimulation of immunoglobulin (Ig) secretion by B-lymphocytes (11). In particular, IL-6 induces bone resorption by itself and in conjunction with other bone-resorbing agents (12). IL-8, formerly known as neutrophil-activating peptide-1 (NAP-1), is important in the initiation and development of inflammatory processes through its capacity to attract and activate neutrophils (13). IL-8-mediated chemotactic and activation effects on neutrophils in the inflamed gingiva may contribute to the periodontal tissue destruction (14). TNF-α, secreted predominantly by monocytes and macrophages, is a potent inflammatory cytokine that upregulates the production of collagenases, prostaglandin (PG) E2, chemokines and cytokines, cell adhesion molecules and bone resorption-related factors (10,14).

The levels of IL-6, IL-8 and TNF-α have been observed to be elevated in chronically inflamed gingival tissues, as well as in the gingival crevicular fluid from patients with periodontitis (8,10,14–18). It has been suggested that the protein expression levels of IL-6, IL-8 and TNF-α may be clinical parameters of gingival and periodontal inflammatory conditions. The aim of the current study was to quantify IL-6, IL-8 and TNF-α levels and assess the correlation of these inflammatory markers in the human gingival tissues of patients with periodontitis.

## Subjects and methods

### Study subjects and Institutional Review Board (IRB)

This study was conducted according to the guidelines of the Declaration of Helsinki and approved by the IRB of the Kyung Hee University Dental Hospital (Reg. no. 2009-1), Seoul, Korea. The participants voluntarily provided written informed consent. Nineteen patients with periodontitis, aged 25–70 years old (49.2±12.7 years, mean age ± standard deviation) were enrolled for the study, which was performed using the gingival tissues of the 19 patients. Samples were obtained whilst the patients underwent periodontal surgery. The tissues of the periodontitis lesions were collected by periodontology specialists in the Department of Periodontology of the Kyung Hee University Dental Hospital.

### Total protein extraction from the tissues of patients with periodontitis

Each sample was homogenized in 500 ml of phosphate-buffered saline (PBS; 137 mM NaCl, 10 mM Na_2_HPO_4_ and 2.7 mM KCl; pH 7.3) with a protease inhibitor cocktail (Roche Korea, Seoul, Korea). The samples were centrifuged at 16,000 × g for 15 min at 4°C, and the supernatant was refrigerated at −70°C until tested. A Bio-Rad protein assay (Bio-Rad Life Science Group, Hercules, CA, USA) was used to quantify the protein concentration.

### Enzyme-linked immunosorbent assay (ELISA)

The assessment of the IL-6, IL-8 and TNF-α levels in the tissues was performed by ELISA, using a human IL-6, IL-8 and TNF-α ELISA Max™ Set Deluxe (BioLegend, Inc., San Diego, CA, USA), in accordance with the manufacturer's instructions. Briefly, one day prior to running the assay, 96-well plates were coated with the capture antibody. Following 18 h incubation at 4°C, the plates were washed with PBS containing 0.05% Tween-20 (Sigma-Aldrich, St. Louis, MO, USA) and then incubated for 1 h at room temperature with a diluent buffer to block nonspecific binding. After washing, 100 ml sample (100 mg) was added to each well and then incubated for 2 h at room temperature. After washing of the plates, 100 ml biotinylated detection antibody was added to each well. The plates were then incubated for 1 h, prior to further washing. Following this, 100 ml avidin-horseradish peroxidase (HRP) was added to each well followed by incubation for 30 min at room temperature. After further washing, 3,3′,5,5′-tetramethylbenzidine (TMB) substrate solution was added and the plates were incubated in the dark for 15 min. The reaction was stopped by the addition of 100 ml 2 N sulfuric acid, and the absorbance at 450 nm and 570 nm was measured.

### Statistical analysis

Statistical analysis for the correlation line was performed using SPSS 20.0 statistical software (SPSS, Inc., Chicago, IL, USA). The Pearson correlation coefficient (r) and associated probability (P) were calculated for the data sets of each combination of the three cytokine expressions. P<0.05 was considered to indicate a statistically significant difference (19).

## Results

The study employed human gingival tissue samples from 19 patients (male, n=14; female, n=5) with periodontitis. The protein expression levels of IL-6, IL-8 and TNF-α were determined by ELISA. As shown in Table I, the protein expression of the three target cytokines was detected in all the gingival tissue samples, with particularly high levels of IL-8 observed, compared with those of the other two cytokines.

**Figure 1:**
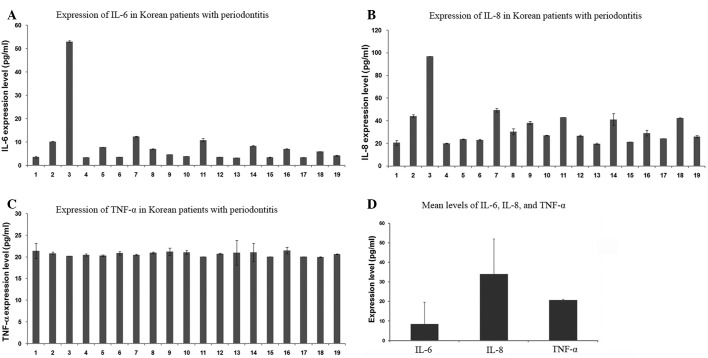
Protein levels of: (A) interleukin (IL)-6, (B) IL-8 and (C) tumor necrosis factor-α (TNF-α) in each patient with periodontitis. (D) Mean levels of IL-6, IL-8 and TNF-α in the gingival tissues of 19 patients. Protein levels were evaluated by enzyme-linked immunosorbent assay (ELISA), and three triplicate experiments were independently performed. Data are presented as the mean ± standard deviation.

In Table I and Fig. 1, the concentrations of IL-6, IL-8 and TNF-α in the gingival tissue samples from the 19 patients with periodontitis are presented. The IL-6 levels of the majority of the patients were observed to measure between 3 and 13 pg/ml, with the exception of one patient (No. 3, 53.08±0.45 pg/ml; Fig. 1A). Similarly, the IL-8 levels were detected to be between 19 and 50 pg/ml, with the exception of one patient (No. 3, 97.04±0.01 pg/ml; Fig. 1B). However, the TNF-α levels in all samples were between 20 and 22 pg/ml (Fig. 1C). Interindividual fluctuations were observed in the IL-6 and IL-8 levels among the 19 patients, while there was little difference in the TNF-α levels (Table I). As shown in Fig. 1D, the mean levels of IL-6, IL-8 and TNF-α were 8.41±0.25, 34.01±1.09 and 20.70±0.31 pg/ml, respectively. In a comparison of the three tested cytokines, IL-8 was highly expressed, whereas IL-6 was detected at low levels and TNF-α was moderately expressed. The mean levels of IL-6, IL-8 and TNF-α in the 14 male patients were 9.69, 35.93 and 20.78 pg/ml, respectively, and in the five female patients were 4.80, 28.63 and 20.47 pg/ml, respectively (data not shown). Although the female sample number was small, the mean levels of IL-6 and IL-8 in the five female patients appeared to be lower than those in the 19 patients as a whole, while the mean level of TNF-α in the five female patients appeared consistent with that of the 19 patients. The mean level of IL-6 in the five female patients (4.80 pg/ml) was ~49.5% of that in the 14 male patients (9.69 pg/ml), which was due to the fact that the interindividual fluctuations of the IL-6 levels in the female patients were relatively small, and the levels remained between 3 and 8 pg/ml.

**Figure 2:**
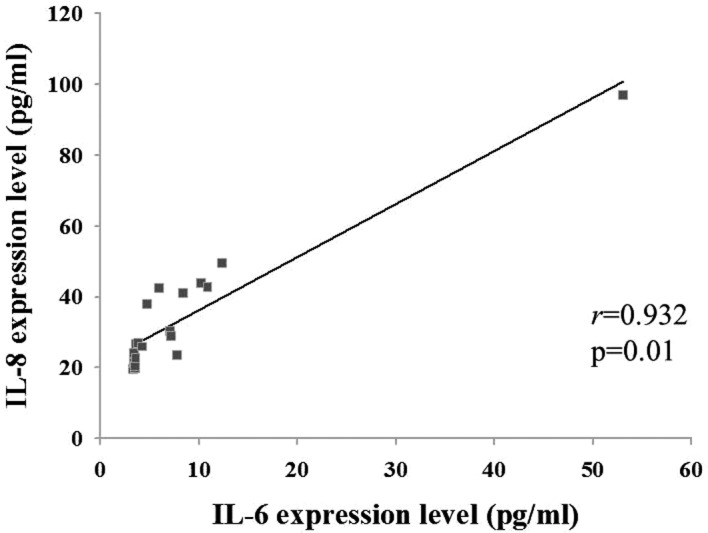
Correlation between interleukin (IL)-6 and IL-8 levels, assessed by the Pearson correlation coefficient (r) and associated probability (p).

As shown in Fig. 2, the Pearson correlation coefficient (r) and associated probability (P) were assessed for the levels of the three cytokines. The IL-6 and IL-8 levels were positively correlated with each other (r=0.932, P=0.01); however, the TNF-α level was not correlated with the IL-6 or IL-8 levels (data not shown).

## Discussion

Periodontal diseases may lead to chronic inflammatory conditions and the destruction of the supporting structures of the dentition. Periodontal diseases are caused by gram-negative bacterial infections, which produce lipopolysaccharides (LPS) (1). The bacterial infection and inhabitation is the etiological factor of periodontitis, due to the fact that the host response and immune reaction to such infectious organisms mediate the development of the periodontitis, rather than infection itself (1). Previous studies have revealed the roles of TNF-α (5), IL-1β (14) and IL-6 and IL-8 (20) as proinflammatory cytokines. Consistent with other inflammatory diseases, proinflammatory cytokines, including IL-6, IL-8 and TNF-α, appear to be major mediators in periodontitis (14). Therefore, in the current study, the levels of IL-6, IL-8 and TNF-α expressed in the human gingival tissues of patients with periodontitis were investigated.

IL-6 is produced by a number of different cell types, such as monocytes, macrophages, fibroblasts, endothelial cells, epithelial cells, T- and B-cells and keratinocytes. IL-6 is also expressed in a variety of situations involving host immune responses and inflammatory reactions (10). With regard to periodontal diseases, an immunohistochemical study observed that a higher level of IL-6 was expressed in inflamed gingival tissues than in healthy control tissues (21). Moreover, previous data obtained using a reverse transcription polymerase chain reaction (RT-PCR) and ELISA showed that the mRNA expression, as well as protein expression, of IL-6 was increased in patients with periodontal diseases (22,23). The data from the present study revealed that IL-6 was expressed in the gingival tissue of each of the 19 patients with periodontitis. The results from the present study for the IL-6 levels, obtained using ELISA, were consistent with previous findings.

IL-8 is an established chemotactic protein and is released in inflamed human gingival tissues (10,24). Gingival fibroblasts affected by infectious organisms express higher levels of IL-8 mRNA in comparison with the baseline expression (23). Our data showed that IL-8 was highly expressed in the gingival tissues of all the patients with periodontitis. The present results were consistent with those from previous studies. However, the mean level of IL-8 was higher than that of IL-6 or TNF-α. Moreover, as shown in Fig. 2, the IL-8 level was positively correlated with the IL-6 level in each sample. The results suggested that IL-8 may be important in the identification of patients with periodontitis.

TNF-α is considered to be a major cytokine involved in the pathogenesis of periodontal disease, affecting the consequences of the tissue destruction and the erosive reaction in periodontitis (25). TNF-α is produced by monocytes and macrophages in response to oral bacterial components, such as LPS. Elevated levels of TNF-α may promote the release of collagenase from human gingival fibroblasts (10), leading to cartilaginous collagen destruction and bone resorption (25). It has been demonstrated that there is no correlation between the level of TNF-α and representations of chronic degenerative changes, such as gingival index, plaque index or probing depth (26). Therefore, it has been suggested that TNF-α may be a marker of inflammatory activity. In the present results, the concentration of TNF-α showed little difference among the 19 patients, which contrasted with the fluctuations of the IL-6 or IL-8 levels. Furthermore, the TNF-α expression did not correlate with the IL-6 or IL-8 levels. The results suggest that ILs and TNF-α may be expressed through different pathways in the pathophysiology of periodontitis.

In conclusion, the levels of three cytokines (TNF-α, IL-6 and IL-8) in the human gingival tissues of 19 patients with periodontitis were detected by ELISA. The mean levels of TNF-α, IL-6 and IL-8 were 8.41±0.25, 34.01±1.09 and 20.70±0.31 pg/ml, respectively. The expression of the two ILs (IL-6 and IL-8) was revealed to be positively correlated (r=0.932, P=0.01). It was suggested that the increased levels of these inflammatory cytokines in periodontitis may have diagnostic and prognostic potentials for the monitoring of the disease and the therapeutic decisions.

## Figures and Tables

**Table I:** Demographic and laboratory data of the 19 patients with periodontitis.

No.	Gender	Age (years)	IL-6 (pg/ml)	IL-8 (pg/ml)	TNF-α (pg/ml)
1	M	49	3.53±0.42	20.49±2.24	21.42±1.74
2	M	54	10.21±0.07	44.06±1.17	20.87±0.32
3	M	56	53.08±0.45	97.04±0.01	20.28±0.01
4	M	70	3.47±0.02	19.98±0.33	20.51±0.23
5	M	31	7.80±0.03	23.54±0.37	20.32±0.17
6	M	64	3.56±0.03	22.85±0.67	20.87±0.35
7	M	54	12.34±0.15	49.50±1.64	20.50±0.13
8	M	42	7.02±0.24	30.40±2.70	20.96±0.18
9	M	47	4.72±0.04	38.13±1.06	21.24±0.86
10	M	47	3.84±0.01	26.88±0.29	21.09±0.39
11	M	63	10.86±0.75	42.94±0.01	20.05±0.01
12	M	53	3.60±0.01	26.71±0.58	20.78±0.09
13	M	39	3.31±0.01	19.53±0.63	20.98±2.86
14	M	63	8.35±0.23	41.06±5.41	21.07±2.08
15	F	42	3.41±0.15	21.40±0.01	20.09±0.01
16	F	65	7.12±0.01	28.99±2.51	21.52±0.77
17	F	25	3.40±0.04	24.23±0.01	20.06±0.01
18	F	32	5.89±0.03	42.53±0.20	20.02±0.09
19	F	40	4.20±0.25	26.00±0.92	20.66±0.06

All proteins were normalized per 100 μg protein sample. Three triplicate experiments were independently performed. Data are presented as the mean ± standard deviation. IL, interleukin; TNF-α, tumor necrosis factor-α; M, male; F, female.
